# Mannan-Binding Lectin in Cardiovascular Disease

**DOI:** 10.1155/2014/616817

**Published:** 2014-04-30

**Authors:** Izabela Pągowska-Klimek, Maciej Cedzyński

**Affiliations:** ^1^Department of Anesthesiology and Intensive Care, Polish Mother's Memorial Hospital Institute, Rzgowska 281/289, 93-338 Łódź, Poland; ^2^Laboratory of Immunobiology of Infections, Institute of Medical Biology, Polish Academy of Sciences, Lodowa 106, 93-232 Łódź, Poland

## Abstract

Cardiovascular disease remains the leading cause of mortality and morbidity worldwide so research continues into underlying mechanisms. Since innate immunity and its potent component mannan-binding lectin have been proven to play an important role in the inflammatory response during infection and ischaemia-reperfusion injury, attention has been paid to its role in the development of cardiovascular complications as well. This review provides a general outline of the structure and genetic polymorphism of MBL and its role in inflammation/tissue injury with emphasis on associations with cardiovascular disease. MBL appears to be involved in the pathogenesis of atherosclerosis and, in consequence, coronary artery disease and also inflammation and tissue injury after myocardial infarction and heart transplantation. The relationship between MBL and disease is rather complex and depends on different genetic and environmental factors. That could be why the data obtained from animal and clinical studies are sometimes contradictory proving not for the first time that innate immunity is a “double-edge sword,” sometimes beneficial and, at other times disastrous for the host.

## 1. Mannan-Binding Lectin: An Overview of Structure and Synthesis

Historically, innate immunity was identified as the first-line defense system, protecting an organism from invading pathogens and abnormal self-derived components. Its immediate response prevents the spreading of progressive systemic infection after contact with pathogens. Furthermore, it takes part in the clearance of damaged cells and tissues, preventing the development of chronic inflammation, cancer, or uncontrolled autoimmune responses. There are two phases of the innate immune response: recognition and elimination of targets. The innate immunity system functions* via* a network of cellular and humoral factors. Mannan-binding lectin (MBL), also known as mannose-binding lectin or mannan- (mannose-) binding protein (MBP), is a soluble (humoral) pattern-recognition molecule thought to be an important component of the innate immune system. MBL has opsonic activity and, in cooperation with MBL-associated serine proteases (MASPs), the ability to activate complement* via* the lectin pathway.

Mannan-binding lectin belongs to the collectin family, a group of C-type lectins also possessing a collagen-like helical domain. Human MBL exists as a series of oligomers of 2–6 subunits, built up from three identical polypeptide chains (24 kDa, 228 amino acids each). The apparent molecular weights of these oligomers range from approx. 150,000 to approx. 450,000, taking into account glycosylation. It forms a “bouquet-like” structure. MBL, like other collectins, is characterized by the presence of four regions. (1) The short N-terminal cysteine-rich domain is responsible for the arrangement of subunits in the oligomer, dependent on disulphide bonds; this region consists of 21 amino acids, including three Cys residues. (2) The collagen-like region interacts with MASPs; it consists of 59 amino acids (among them 19 Gly-X-Y triplets); this domain is glycosylated. (3) Next, an *α*-helical “neck” region (30 amino acids) stabilizes polypeptide chains within a structural subunit. (4) The C-terminal carbohydrate-recognition domain (CRD) is responsible for pattern recognition and consists of 118 amino acids [1–6]. MBL binds with the highest affinity to D-mannose, N-acetyl-D-glucosamine, and L-fucose which allows the specific recognition of numerous polysaccharides and glycoconjugates like bacterial lipopolysaccharides, capsular polysaccharides, fungal mannans, and so forth. It moreover recognizes some phospholipids,* Neisseria* outer membrane proteins, and DNA of apoptotic cells [7–9]. Mannan-binding lectin is synthesized by hepatocytes and secreted into the blood in an oligomeric form. Moreover, specific mRNA has been found in bone marrow, fetal lung, small intestine, and testis [10]. Its synthesis is controlled by the* MBL2* gene, located on chromosome 10 (10q11.2) and regulated in a similar manner to acute phase proteins. The plasma concentration of MBL can increase up to 3-fold in response to infection. In healthy individuals, an average plasma (serum) level is approx. 1 *μ*g/mL; however it ranges from <0.1 *μ*g/mL up to >5 *μ*g/mL. The* MBL2 *gene contains 4 exons and 3 introns. Most mammals (but not humans or chimpanzees) synthesize two MBL forms: A and C. Human MBL resembles the C form. The* MBL1 *pseudogene (not expressed), corresponding to the A form, has been found in the human genome. Exon 1 of the* MBL2* gene encodes the signal peptide, the cysteine-rich domain, and part of the collagen-like region. Exon 2 encodes the remaining part of the latter. Exon 3 encodes the neck region while exon 4 encodes the CRD [3–6, 11, 12].

MBL deficiency, believed to be the most common human immunodeficiency, markedly depends on* MBL2* gene point mutations in codons 52, 54, and 57 of exon 1. These variants, giving dominant alleles D, B, and C, respectively (commonly designated collectively as O; the wild-type is designated as A), lead to disruption of the collagen domain structure. This, in consequence, prevents oligomerization of the basic triplet polypeptide subunit (and therefore normal interaction with MASPs) resulting in diminished complement activation and opsonic activity [6, 13–15].

A shortened biological half-life of the protein is a reflection of increased sensitivity to serum metalloproteases [16]. As well as the aforementioned mutations, polymorphisms in the promoter region (at positions –550 and –221; variants H/L and Y/X, resp.) and the untranslated region of exon 1 (at position +4, variants P/Q) have been described. The first two (and possibly the third) influence gene expression and, in consequence, the serum concentration of the protein. The highest MBL level occurs in association with promoter genotype HYP/HYP, and the lowest with LXP/LXP homozygotes [6, 13–15].

## 2. MBL-Dependent Complement Activation

Until recently, MBL was believed to be the sole collectin able to activate the lectin pathway (LP) of complement. However, several reports indicate that MBL shares that property not only with ficolins (another family of collagen-related lectins) but also with the so-called “novel collectins,” like collectin 11 (CL-11 or collectin-kidney 1, CL-K1) and collectin 10 (CL-10 or collectin-liver 1, CL-L1) [17, 18]. After binding of the MBL-MASP complex to the target structure, conformational changes lead to the activation of MASPs which in consequence makes the cleavage of C4 and C2 possible and thus the initiation of the complement cascade. In spite of the different initiation mechanism, the lectin pathway resembles the classical pathway (CP), forming the same convertases for C3 and C5 components. Within the MASP family, three proteases (MASP-1, MASP-2, and MASP-3) and two related, nonenzymatic proteins, MAp19 (sMAP) and MAp44 (MAP-1), have been identified. MASP-1, MASP-3, and MAp44 are the products of alternative splicing of the* MASP1/3* gene while MASP-2 and MAp19 are synthesized under the control of the* MASP2* gene [4, 19–21].

**Figure 1:**
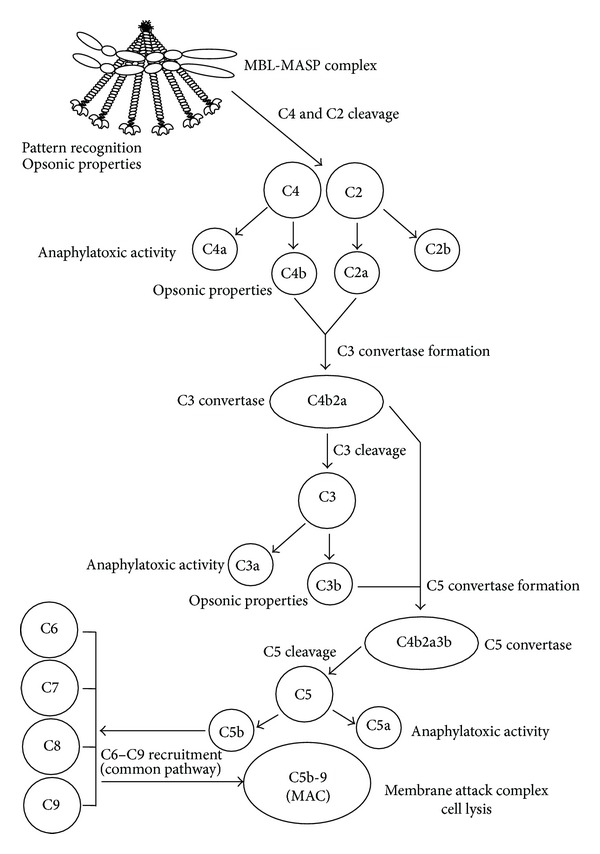
Schematic overview of MBL-dependent activation of the lectin pathway of complement. Modified from [5]. Scheme of MBL-MASP complex based on [23].

MASP-2 is believed to be the key enzyme responsible for LP activation as its proteolytic activity against C4 and C2 significantly exceeds the activity of C1s, the corresponding element of the classical pathway [4, 19, 22]. MASP-1 was believed to upregulate lectin pathway activation. However, recently, its crucial role in MASP-2 activation has been postulated [23–25]. MASP-3 and nonenzymatic proteins are believed to play a regulatory role in this process [19, 26–28]. A schematic overview of LP activation is presented in Figure 1.

## 3. Involvement of MBL-Associated Serine Proteases in Coagulation and Kallikrein-Kinin Systems

The increased infection susceptibility of MBL-deficient individuals is now well documented by numerous laboratory and clinical data but its description is outside the scope of this review. Recent studies bring an increasing amount of evidence that MBL plays an important role in other systemic processes including coagulation, inflammation, and tissue injury.

MASP-1 and -2 may participate in activation of the coagulation system. The former is able to cleave fibrinogen, factor XIII, and thrombin-activatable fibrinolysis inhibitor (TAFI) [29–34] while MASP-2 is able to cleave prothrombin [32]. Moreover, Megyeri et al. [35] found MASP-1 to interact with protease-activated receptor-4, a mediator of inflammation and platelet activation. Later, Dobó et al. [36] found high-molecular-weight kininogen to be its additional substrate. This activity (like that of kallikrein) enables release of bradykinin, a highly proinflammatory mediator of the kinin-kallikrein system. Although MASP-2 cleaves kininogen as well, no bradykinin is released during this process [36].

The contribution of MASPs to fibrin clot formation on the surface of pathogens may limit dissemination of infection [32]. The involvement of MBL-MASP complexes in haemostatic processes was later demonstrated in an animal model by Takahashi et al. [33] who found that MBL-null mice infected with* Staphylococcus aureus *were predisposed to the development of disseminated intravascular coagulation (DIC). Moreover, MBL- and MASP-1/3- (but not MASP-2- or C3-) null mice had prolonged bleeding time after injury [33].

## 4. MBL in Injury to Peripheral Organs

The impact of components of the lectin pathway (especially MBL) on both systemic inflammation and peripheral organ injury has been extensively investigated. These studies, concerning various clinical conditions, often lead to contradictory conclusions, proving not for the first time that the innate immune response is a “double-edge sword,” sometimes beneficial and at other times disastrous for the host. MBL and MBL-dependent complement activation have been found to be involved in ischaemia-reperfusion (I/R) injury associated with numerous clinical conditions such as graft (kidney, lung) rejection and other pathological processes in the gastrointestinal tract or central nervous system.

### 4.1. Kidney Injury

Significant deposition of MBL-MASP-2 complexes was found in porcine kidney after I/R injury. Moreover, colocalization of C4d with MBL (as with C1q) was observed [37]. Administration of C1 esterase inhibitor (C1-INH) resulted in inhibition of apoptosis of tubular epithelial cells and tubular damage. Therefore, a pathogenic role of both lectin and classical pathways in I/R renal injury was implicated. Møller-Kristensen et al. [38] found that MBL-double knockout (lacking both MBL-A and -C) mice were protected from experimental renal ischaemia-reperfusion damage. Reconstitution with recombinant human MBL led to kidney injury, in a dose-dependent manner. These authors demonstrated involvement of complement activation in this process. Earlier, de Vries et al. [39] found colocalization of MBL with complement factor C6 in the later reperfusion phase. However, contradictory results have recently been published by Miwa et al. [40], who, after crossing decay-accelerating factor (DAF, CD55) and CD59 double knockout mice with animals deficient with various complement components/receptors, demonstrated a crucial role for the alternative but not the classical or lectin pathways. Furthermore, van der Pol et al. [41], using another rodent (rat) model, suggested that, although MBL took part in kidney dysfunction, it was not associated with complement activation. In their study, therapeutic inhibition of MBL was protective against tubular damage, preventing accumulation of macrophages and neutrophils as well as expression of proinflammatory cytokines and chemokines. Following reperfusion, MBL was internalized into tubular epithelial cells, inducing rapid cell death. Therefore, it was concluded that MBL-mediated (but lectin pathway-independent) cytotoxicity preceded complement activation and was the primary reason for tubular injury. Interestingly, a similar effect was demonstrated on human epithelial cells* in vitro* [41].

MBL deposition in ischaemically injured human kidney was first reported by de Vries et al. [39]. It appeared early after transplantation, in peritubular capillaries and tubular epithelial cells. Berger et al. demonstrated a beneficial effect of low (<400 ng/mL) serum MBL concentrations and “low MBL” variants (XA/O, O/O) of the corresponding gene in recipients of kidney or combined pancreas-kidney transplants on graft and patient survival. Low levels of circulating MBL correlated with improved long-term graft survival [42, 43]. In contrast, Bay et al. [44] found the 5-year death-censored renal graft survival to be lower in MBL-deficient patients. In other reports, no impact of polymorphisms of the* MBL2 *gene in recipients [45] or of* MBL2, MASP2, *and* FCN2 *genes (the last mentioned one is responsible for synthesis of another lectin pathway activating molecule, ficolin-2) in either donors or recipients [46] on kidney transplant outcome was observed. Such contradictory results might reflect a dual role of MBL in renal transplantation. Damman and Seelen [47] suggested that under moderate graft injury, lectin pathway activation may be beneficial due to participation in the clearance of dying cells. However, under severe injury, MBL might contribute to the renal tubular epithelium damage.

According to Osthoff et al. [48], MBL deficiency could offer some protection from I/R radiocontrast-induced kidney injury. In patients with serum MBL levels <500 ng/mL, they observed less organ dysfunction.

### 4.2. Cerebral Ischaemia

Several reports demonstrated an association of MBL with cerebral ischaemia. Ducruet et al. [49] observed deposition of MBL (both A and C forms) in murine ischaemic endothelium. In double knockout mice, C3 activation and subacute accumulation of mononuclear cells in the ischaemic region were absent. However, despite improved outcome within one day, the neuroprotective effect was not sustained. Morrison et al. [50] found a beneficial effect of MBL deficiency in striatum only. Even in MBL-null mice, they observed C3 deposition in the hemisphere, during reperfusion, suggesting involvement of other complement activation pathways in the pathology. Later, Orsini et al. [51], working with mice, confirmed MBL deposition in ischaemic vessels and a protective effect of genetically determined MBL deficiency; they also found an increase in circulating MBL-MASP-2 complex concentration. Moreover, a long-lasting protective effect of anti-MBL-A antibody and synthetic MBL inhibitor in rats was noted [51]. Elvington et al. [52] did not detect C3d deposition in C1q- and MBL-deficient mice and found them to be protected from experimental I/R injury. These authors also demonstrated involvement of the third (alternative) pathway of complement activation. Interestingly, C6 deficiency had no effect, indicating the lack of significance of the common pathway and thus forming of the MAC.

Cervera et al. [53] provided data from both animal and clinical studies. The authors induced brain ischaemia by 2-hour occlusion of the middle cerebral artery in MBL-null and wild-type mice (control). MBL-null mice presented smaller infarctions, better functional outcome, and diminished C3 deposition and neutrophil infiltration. However, animals that received recombinant human MBL showed significantly larger areas of infarction. In the clinical study, an unfavourable outcome in stroke patients (109 ischaemic, 24 haemorrhagic) after 3 months was associated with MBL-sufficient genotypes and higher circulating MBL levels. Based on both studies, the authors concluded that genetically determined MBL deficiency is associated with a better outcome after acute stroke.

Similar results were obtained by Osthoff et al. [54] who determined MBL concentrations in 353 consecutive patients with ischaemic stroke, of whom 287 received conservative while 66 received thrombolytic treatment. Within the latter group, additional samples were analyzed 24 and 72 hours after admission. In the conservatively treated group, with mild strokes, small infarction volume and favorable outcomes after three months demonstrated 1.5–2.6-fold lower median MBL levels compared with patients with more severe strokes. MBL-deficient patients (<100 ng/mL) had significantly lower risk of unfavorable outcome and showed smaller lesion volumes. In the thrombolysis group no association of MBL concentration with infarction volume or functional outcome was found. The authors suggested that inhibition of lectin pathway may be a promising strategy for reducing I/R associated cerebral damage.

In a recent clinical study Wang et al. [55] assessed serum levels of MBL in 148 Chinese patients with acute ischaemic stroke (the blood samples were obtained within 24 hours from recognition). MBL levels were significantly higher in stroke patients compared with healthy controls and increased with increasing severity of stroke. The authors postulated that elevated MBL levels could be an independent stroke risk factor in the Chinese population. However, no data concerning* MBL2 *genotypes were provided.

### 4.3. Lung Transplantation

Several papers have focused on the role of MBL in lung transplantation. Its higher levels in plasma of recipients were associated with development of bronchiolitis obliterans syndrome (BOS) and poorer long-term outcome [56, 57]. Immunohistochemistry revealed the presence of MBL in lung tissue from patients with BOS and at the time of ischaemia [57]. Moreover, Carroll et al. [58] observed a significant increase of MBL concentration in plasma at 3, 6, and 12 months after transplantation. Interestingly, the presence of MBL in bronchoalveolar lavage (BAL) (3 and 6 months after transplant) was associated with later BOS development [58]. In contrast, Hodge et al. [57] found no correlation between blood MBL levels and time after transplantation. They found lower MBL concentrations in BAL from recipients who developed BOS, in comparison with controls and patients with stable graft function. In another study, Kwakkel-van Erp et al. [59] did not report any impact of MBL levels on BOS but they noted a significant decrease of serum MBL after transplantation. Low MBL concentrations were, however, associated with a longer survival. Earlier, Munster et al. [60] postulated a beneficial effect of donor's* MBL2 *X gene variant (promoter, position −221) on lung transplant outcome (better graft and BOS-free survival). However, carrying the same allele in hepatitis-C virus- (HCV-) positive recipients of liver transplants was a risk factor for acute cellular rejection [61].

### 4.4. Gastrointestinal Ischaemia

Interesting data have been provided concerning I/R damage in the gastrointestinal tract on mice model. Zhang et al. [62] found that IgM, bound to antigens in ischaemic mesenterium, provided a binding site for MBL resulting in complement activation. This finding was further confirmed by Lee et al. [63] who detected MBL (as well as C1q) complexed to antigen and natural IgM in the intestinal I/R injury model. They, moreover, excluded any involvement of the alternative pathway. That seems to be in agreement with results published by Busche et al. [64], concerning myocardial infarction (see below). Later, Schwaeble et al. [65] postulated MASP-2 deficiency to protect mice from gastrointestinal ischaemia-reperfusion damage.

## 5. MBL in Cardiovascular Disease

### 5.1. MBL in Cardiac Ischaemia-Reperfusion Injury

Involvement of MBL in coagulation and ischaemia-reperfusion (I/R) injury is potentially harmful in the development of cardiovascular disease, especially coronary artery disease and myocardial infarction (MI), as well as in the rejection of a heart transplant.

As coronary artery disease (leading to myocardial infarction) is the leading cause of morbidity and mortality worldwide, one of the therapeutic challenges of modern cardiology is to create a strategy to reduce the area of infarction and improve cardiac repair after MI. That is why research continues into the mechanisms of cardiac ischaemia-reperfusion injury and possibilities of interventions. Ischaemia changes expression of surface molecules and leads to formation of neoantigens. Paradoxically, reperfusion causes a harmful inflammatory response that can counteract the beneficial effects of improved blood flow. During reperfusion, myocardial cells become the targets of innate immunity: promoting release of inflammatory mediators, neutrophil recruitment, oxidative stress, complement, and TLR activation [66].

#### 5.1.1. Data from Animal Models

The possible impact of lectin pathway factors on myocardial ischaemic injury was first demonstrated by Collard et al. [67] who observed MBL deposition in ischaemic rodent heart. Their* in vitro* experiments proved moreover that endothelial iC3b deposition after oxidative stress was attenuated by MBL ligands (as N-acetyl-D-glucosamine or D-mannose) as well as in MBL-deficient serum. Later, Jordan et al. [68] showed that the blocking of MBL-dependent LP activation reduced the extent of myocardial ischaemia-reperfusion injury in rats. Furthermore, Walsh et al. [69] found that MBL-deficient mice were protected from cardiac ischaemic injury. In contrast, in animals lacking C1q (but with intact MBL), no protective effect was noted. The importance of the lectin pathway was confirmed by Schwaeble et al. [65] who demonstrated that MASP-2-deficient, but not C4-deficient, mice had a significant reduction in myocardial infarct size. That moreover suggested that LP may be activated* via *C4-by-pass, as LP-mediated C3 activation required MASP-2, MASP-1/3, and C2. Recently, Pavlov et al. [70] showed that inhibition of the lectin pathway using MAP-1 (MAp44) preserved cardiac function, decreased infarct volume, decreased C3 and MBL deposition, and prevented thrombogenesis.

An interesting insight into the mechanisms causing myocardial ischaemia-reperfusion (MI/R) injury was provided by Busche et al. [64]. Using MBL-A-, MBL-C-, and secreted IgM-null mice, they found that myocardial tissue injury following MI/R, associated with complement activation, depended on both MBL and IgM antibodies. That seems to be in agreement with the results published by Zhang et al. [62] and Lee et al. [63], concerning gastrointestinal IR previously mentioned. Earlier, the same group suggested MBL played a critical role in MI/R connected with diabetes [71].

#### 5.1.2. Clinical Data

As in experimental animal models, the role of MBL in cardiovascular disease has been investigated in clinical settings. Pesonen et al. [72] found lower serum concentrations of C3, higher levels of MBL, and a higher frequency of high MBL level-associated* MBL2 *genotypes in a cohort of patients with unstable angina pectoris or acute myocardial infarction compared with healthy controls. Similarly, Haahr-Pedersen et al. [73] observed significantly higher MBL and lower soluble C5b-9 (complement membrane attack complex, MAC) concentrations in sera of patients with ST-elevation myocardial infarction (STEMI), undergoing primary percutaneous coronary intervention with left ventricular ejection fraction (LVEF) <35% compared with those with LVEF ≥35%. Interestingly, Keller et al. [74] noted that an elevated MBL level is a risk factor for future development of coronary artery disease (CAD) in apparently healthy men but not in women. Recently, Schoos et al. [75] have found high plasma levels of MBL and ficolin-2 (L-ficolin) to be synergistically associated with increased postinfarct left ventricular end systolic and diastolic volumes (ESV, EDV) in STEMI patients. Correspondingly, Trendelenburg et al. [76] reported that MBL functional deficiency (defined as serum concentration ≤100 ng/mL) contributed to the significant reduction of 90-day mortality in patients with acute STEMI, undergoing early reperfusion by catheter revascularization. On the other hand, no association of low MBL with combined endpoint of death, development of shock and congestive heart failure, or laboratory markers of ischaemia (kinase creatinine CK and CK-MB levels) was observed. As the main cause of patients' death was fatal cardiac arrhythmia, the authors speculated that MBL deficiency did not affect the size of infarction but might influence the risk of arrhythmia [76]. Bilgin et al. [77] found that MBL-deficient (serum concentration ≤80 ng/mL) patients with sterile systemic inflammation (systemic inflammatory response syndrome, SIRS) after cardiac surgery did not develop multiple organ failure (MOF) unless MBL was reconstituted by transfusion of fresh frozen plasma. Later, it was hypothesized (although based on data from one person) that postoperatively transfused MBL in MBL-deficient individuals (an increment of serum level from <1 ng/mL up to nearly 400 ng/mL) with acute myocardial infarction might result in a fatal outcome [78]. Recently, Holt et al. [79] demonstrated higher plasma concentrations of* MASP1/3 *gene products (MAp44, MASP-1, and MASP-3) to be higher in patients with MI compared to healthy controls. That was, however, not associated with short-term outcome (salvage index, final infarct).

Several reports, however, provide opposite findings. A clinical prospective study by Saevarsdottir et al. [80] recruited more than 19000 participants and estimated the risk of myocardial infarction in relation to MBL concentration. The influence of such factors as sex, age, diabetes, hypercholesterolemia, hypertension, smoking, and elevated erythrocyte sedimentation rate (ESR) was taken into consideration as well. MBL levels >1 *μ*g/mL (defined by authors as “high”) were associated with decreased risk of MI, independently of other factors. Further analysis of data from the randomly selected nested case-control sample (>1300 persons) revealed that higher MBL markedly decreased MI risk in individuals with diabetes, hypercholesterolemia, or raised ESR (but not in smokers or hypertensive patients). Moreover, there was no difference in a risk of infarction between diabetic and nondiabetic patients having MBL concentrations >1 *μ*g/mL [80]. These results seem to be in agreement with data published by Vengen et al. [81] who found that possession of MBL deficiency-associated genotypes (XA/O, O/O) was associated with double the incidence of MI at middle age (29–62 yrs). Such* MBL2 *gene variants as well as low plasma concentrations of its product were associated with increased frequency of significant artery stenosis. Again, MBL deficiency happened to be the risk factor independent of “conventional” risk factors. Single nucleotide polymorphisms (SNPs) of genes for ficolins did not influence the risk of myocardial infarction.

Mellbin et al. [82] reported the distribution of* MBL2 *gene variant alleles in patients suffering from type 2 diabetes and MI, to be similar to that in the general population. No greater impact of the genotype or corresponding phenotype on the risk of infarction was noted. However, MBL concentration below average in “low-coding MBL genotype” (defined as XA/XA, A/O, or O/O) carriers was suggested to be disadvantageous.

### 5.2. MBL and Chronic Coronary Artery Disease

Numerous papers refer to clinical and experimental studies evaluating the role of MBL in the development of chronic coronary artery disease. Matthijsen et al. [83] found the deposition of MBL but not enhanced gene expression in human ruptured atherosclerotic lesions. They moreover observed that low-density lipoprotein receptor-null mice with MBL-A- and MBL-C-deficient monocytic cells, fed on a high-cholesterol diet, were more likely to develop atherosclerotic lesions than their MBL-A, -C (+/+) counterparts. That could be caused by reduced removal of apoptotic cells and cellular debris in the absence of MBL. Oral fat loading tests in volunteers showed MBL-deficient individuals (<0.42 *μ*g MBL per mL of serum, determined in a functional hemolytic assay) to have higher postprandial lipid values (contributing to the development of atherosclerosis) in comparison with MBL-sufficient controls [84].

The development of atherosclerosis is known to be associated with* Chlamydia pneumoniae* infection. The presence of this microorganism within atheroma has been reported in several studies [85–91]. Involvement of MBL in host defense against* Chlamydia *was originally suggested by Swanson et al. [90], based on inhibition of infection of cell cultures by various bacterial strains with recombinant lectin. Further, Rugonfalvi-Kiss et al. [91] suggested that carriage of O* MBL2* alleles may be associated with development and progression of severe coronary artery disease in* C. pneumoniae*-infected individuals. The role of MBL in pathophysiology of cardiovascular disease in Danish patients with rheumatoid arthritis was considered by Troelsen et al. [92, 93]. They found that high serum MBL concentrations and “high MBL producing” genotypes contributed significantly to the risk of ischaemic heart disease, myocardial infarction, overall death, and death due to cardiovascular disease [92, 93]. Data from patients with Kawasaki disease suggest that the impact of* MBL2 *polymorphism may be age dependent: children younger than 1 year with variant alleles were at higher risk of development of coronary artery lesions than those without, while among older children, the relationship was the opposite [94, 95].

**Figure 2:**
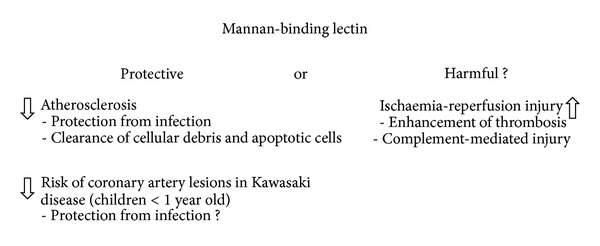
Ambiguous role of mannan-binding lectin in the development of coronary artery disease and myocardial infarction. Based on data reviewed in Section 5.

Data reviewed in this section demonstrate that the influence of MBL on the development of cardiovascular disease and/or prognosis for patients is complex. MBL and activation of the lectin pathway of complement may be protective against the development of atherosclerotic lesions by clearance of apoptotic cells and cell debris from atherosclerotic plaques or by protection from* Chlamydia pneumoniae* infection. On the other hand, MBL may take part in I/R injury processes and enhance thrombosis. In consequence, its resultant effect may depend on diverse factors, specific for the individual, including accompanying diseases, life style, age, and sex. The importance of MBL-dependent mechanisms is still not fully understood. The ambiguous role of MBL in the development of coronary artery lesions and myocardial infarction is presented in Figure 2.

### 5.3. MBL and Heart Transplantation

Similarly, contradictory results concerning the role of MBL in heart transplantation have been published. The long-term success of heart transplantation is limited by the development of cardiac allograft vasculopathy (CAV). CAV is characterized by diffuse and concentric narrowing of the coronary arteries as a result of neointimal expansion that is often accompanied by adventitial fibrosis. It remains one of the major causes of death after transplantation, although the pathogenesis is still not fully understood. A key feature is the development of donor-specific antibodies against human leukocyte antigens (HLA). The severity of CAV correlates with persistent inflammation and a higher degree of HLA mismatch [96–98]. Although immunosuppressive drugs permit successful heart transplantation, they do not always prevent chronic rejection. Innate immune responses like infiltration by NK cells, activation of Toll-like receptors (TLR), and complement deposition are known to participate in the acute rejection of cardiac allografts and in the development of CAV [98]. The involvement of complement in graft rejection was first suggested in 1999, when Baldwin et al. found deposition of C4d and C3 in a series of cardiac biopsy specimens in the first weeks after transplantation and demonstrated that this deposition was associated with the peritransplant ischaemic injury [99]. However, the role of MBL and lectin pathway activation has not been fully elucidated. Fiane et al. [100] showed MBL deficiency (serum level <0.1 *μ*g/mL) to be related to the development of graft-associated coronary artery disease (GACD) and acute rejection episodes after heart transplantation. However, higher complement activity was found in patients with ischaemia (C4bc level reflecting lectin and classical pathways). As there were no differences in concentrations of classical pathway serine proteases C1r/C1s complexed with C1-inhibitor (C1-INH), the authors postulated that the lectin pathway may play a leading role. Moreover, the soluble terminal complex, resulting from all three pathways, correlated with mortality. Later, Fildes et al. [101] did not observe an association of MBL concentrations and GACD, but they reported less acute graft rejection episodes in MBL-deficient patients.

### 5.4. Therapeutic Complement Inhibition in Cardiovascular Disease

Since the role of complement in the pathophysiology of cardiovascular disease (atherosclerosis, myocardial infarction, heart transplant rejection, cardiac surgery complications) was established, a series of studies evaluating the benefits of complement inhibition has been conducted. Apart from the above-mentioned experimental studies employing anti-MBL [67, 68] or anti-MASP-2 antibodies [65], the selective inhibition of the initial steps of the lectin pathway has not been fully investigated and lacks clinical evaluation. Rather, the attention has been turned to a potent complement inhibitor, C1-INH, widely used in the treatment of hereditary angioedema. C1-INH is an acute phase protein, belonging to the serpins, a superfamily of serine protease inhibitors. It has a wide range of biological functions: interacts with coagulation/fibrinolysis and contact systems (inactivates factors XII, XI, thrombin, plasmin, tissue plasminogen activator, and kallikrein), extracellular matrix, circulating neutrophils and macrophages (enhances phagocytosis), endothelial cells (inhibits leukocyte adhesion), infectious agents (bacteria:* Escherichia coli, Bordetella pertussis*; parasites:* Plasmodium falciparum*), and endotoxins (from* Salmonella typhimurium*) and inhibits all pathways of complement activation [102, 103]. It is known to inactivate C1r and C1s (classical pathway) as well as MASP-1 and MASP-2 (lectin pathway). Moreover, C1-INH* in vitro* influences the alternative pathway* via* reversible binding to C3b, thus interfering with C3b-factor B interactions [104, 105].

Since the 1990s, several studies have been published, proving successful use of C1-INH in animal models of myocardial injury. Buerke et al. [106] tested the cardioprotective effect of C1-INH in a feline model of myocardial I/R. C1-INH administration 10 min prior to reperfusion improved recovery of cardiac contractility, decreased leukocyte accumulation, preserved coronary vascular endothelial function, and decreased the amount of necrotic tissue, compared with controls. Six years later, the same group carried out a similar study, using a novel small molecule C1s-inhibitor in a rabbit model of myocardial ischaemia-reperfusion injury that yielded similar conclusions [107]. In Horstick's et al. study, intracoronary application of C1-INH reduced myocardial damage in a porcine model of myocardial ischaemia (60 min of coronary occlusion followed by 2 h of reperfusion) [108]. The same authors paid attention to the importance of properly adjusted dosage of C1-INH [109]. Using the same animal model, they found that administration of 40 IU of an inhibitor per kg reduced myocardial injury; 100 IU/kg did not offer beneficial effects while a large dose (200 IU/kg) provoked side effects and coagulation disorders. In contrast, Schreiber et al. [110] found no influence of C1-INH on the area of infarction or ventricular function in a porcine model.

The beneficial role of C1-INH in limiting ischaemia-reperfusion injury probably is not only the consequence of complement inhibition. Administration of C1-INH in a rat model of MI [111] improved cardiac function and reduced myocardial infarct size due to the inhibition of complement activation and leukocyte recruitment (due to lower expression of the endothelial adhesion molecules, P-selectin, and intercellular adhesion molecule 1 (ICAM-1). Higher doses (100 IU/kg) of C1-INH led to prolonged beneficial effects in comparison with lower (10 or 50 IU/kg) doses. In a recent study by Lu et al. [112], C1-INH-deficient, C3-deficient, and wild-type mice were subjected to coronary artery occlusion (left anterior descending branch) and subsequent reperfusion. C1-INH was administered prior to reperfusion. Besides the cardioprotective effect of C1-INH, a decrease in neutrophil accumulation was observed compared with vehicle-treated animals. This finding seems to confirm the contribution of C1-INH to inhibition of leukocyte recruitment into ischaemic tissue. Another postulated beneficial effect of C1-INH administration after I/R injury is its antiapoptotic activity. In a rat model of MI, C1-INH caused the reversal of the Bcl-2/Bax expression in the myocardial infarct area [113].

Encouraging animal experiences led to clinical application of C1-INH in cardiac disease. In 1998 Bauernschmitt et al. [114] presented their first experiences with C1-INH as a rescue therapy in three patients undergoing emergency surgical revascularization, after failed percutaneous coronary angioplasty, and achieved rapid restoration of myocardial function during reperfusion. In 2002 de Zwann et al. [115] showed that an intravenous bolus followed by an infusion of C1-INH given 6 hours after myocardial infarction in 22 patients caused reduction in levels of CK-MB, troponin T, and circulating C4 fragments. Thielmann et al. [116] studied the effects of C1-INH in patients undergoing emergency coronary artery bypass grafting after acute ST-elevation myocardial infarction. Cardiac troponin I levels were markedly reduced in the study group (*n* = 29), given two C1-INH doses (40 IU/kg during reperfusion and 20 IU/kg, 6 h after surgery), compared with the controls (*n* = 28). Similar results were reported by Fattouch et al. [117], in a larger group of patients (38 persons treated with two doses of 500 IU each, first given before reperfusion, followed by the second, 3 h after surgical intervention, and 28 persons receiving placebo). Despite these promising findings, C1-INH will not be accepted for clinical practice unless it is confirmed by multicenter, randomized trials.

Inhibitors of later stages of complement activation, such as pexelizumab (anti-C5 monoclonal antibody) or TP10 (soluble derivative of human complement receptor type 1 (CR-1), accelerating the decay of C3 and C5 convertases through proteolytic degradation of C3b and C4b) were evaluated in both animal models and clinical trials involving patients after coronary artery bypass graft (CABG) surgery [118–125]. Some benefit was observed in a small group of patients but the primary endpoints were not met in large clinical trials. Description of these studies lies outside the scope of this review.

## 6. Summary and Conclusion

The involvement of complement activation in the pathogenesis of cardiovascular disease and ischaemia-reperfusion injury in general is well documented. However, the role of MBL and the lectin pathway cannot be unequivocally defined, as published data points to opposite and sometimes confusing conclusions (Table 1). It should be remembered that MBL is just one component of the complicated network of complex mechanisms involving host and environmental factors. That is probably the reason why the attempts to influence complement activity therapeutically, although often promising, are still not fully successful.

## Figures and Tables

**Table 1:** Major associations of mannan-binding lectin with myocardial infarction in humans.

Suggested MBL effect	Clinical association	Reference
Harmful	“High MBL” genotypes more frequent and higher levels of MBL in sera of patients with acute MI compared to controls	Pesonen et al. [72]
	Higher levels in patients with STEMI and LVEDF <35% comparing to controls with STEMI and LVEDF >35%	Haahr-Pedersen et al. [73]
	High serum levels associated with increased postinfarct ventricular ESV and EDV in STEMI patients	Schoos et al. [75]
	Low serum levels associated with lower mortality	Trendelenburg et al. [76]

Protective	High serum level associated with decreased risk of MI	Saevarsdottir et al. [80]
	“MBL deficient” genotypes connected with higher likelihood of MI at middle age	Vengen et al. [81]
